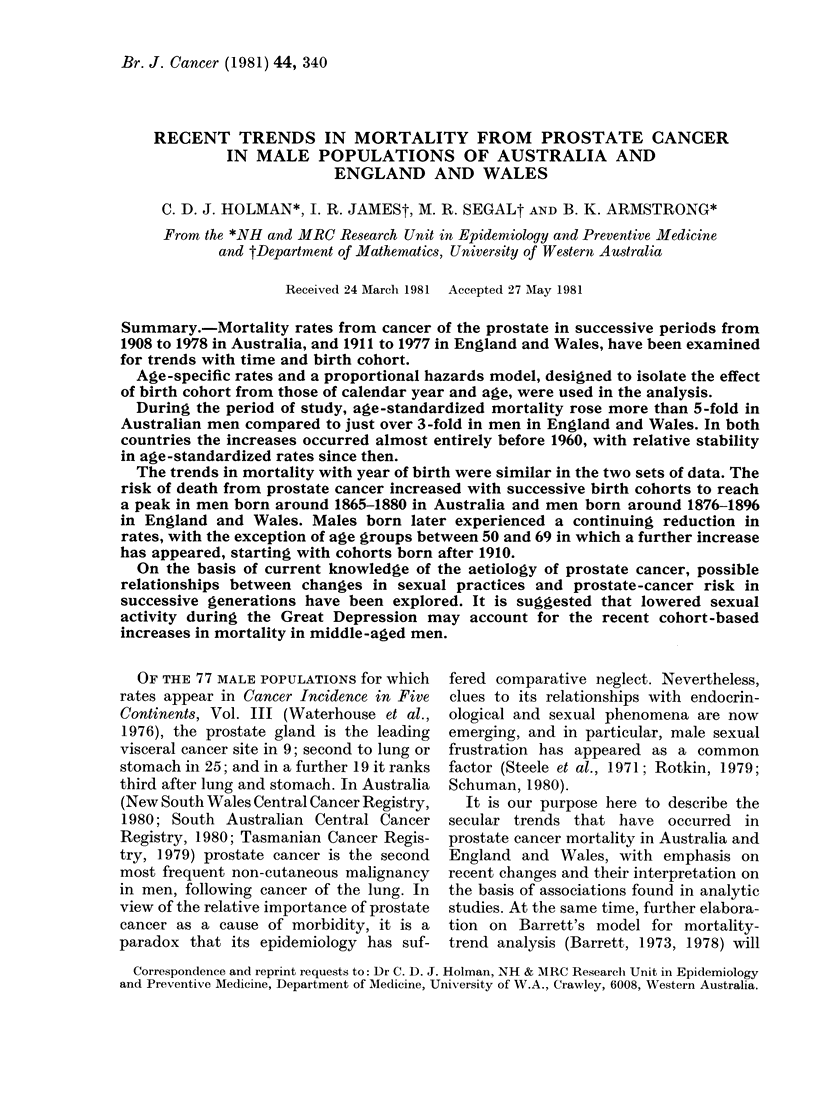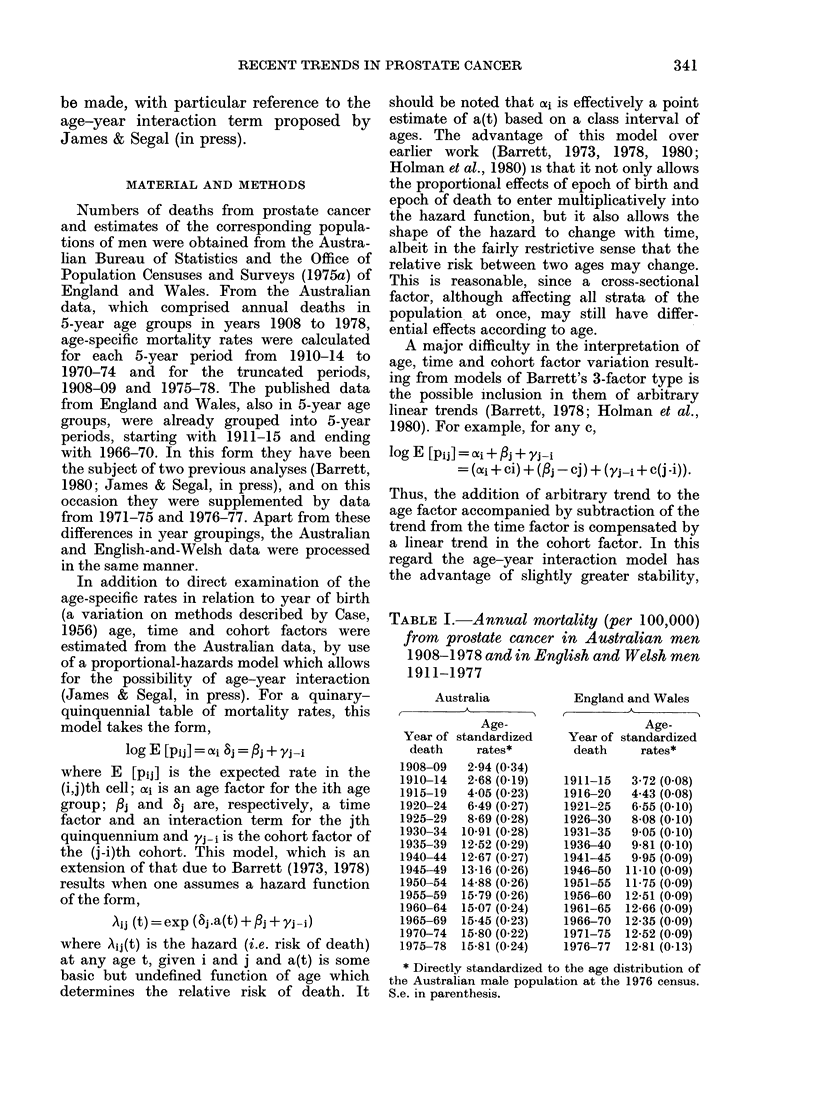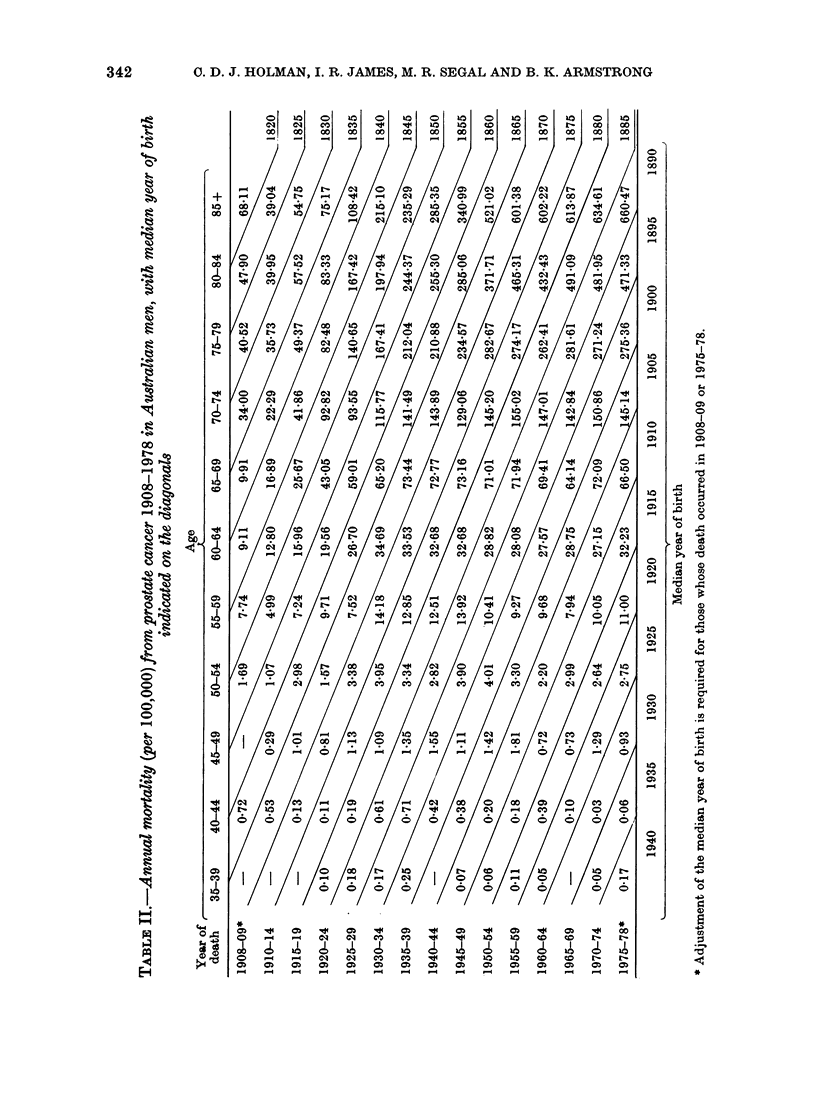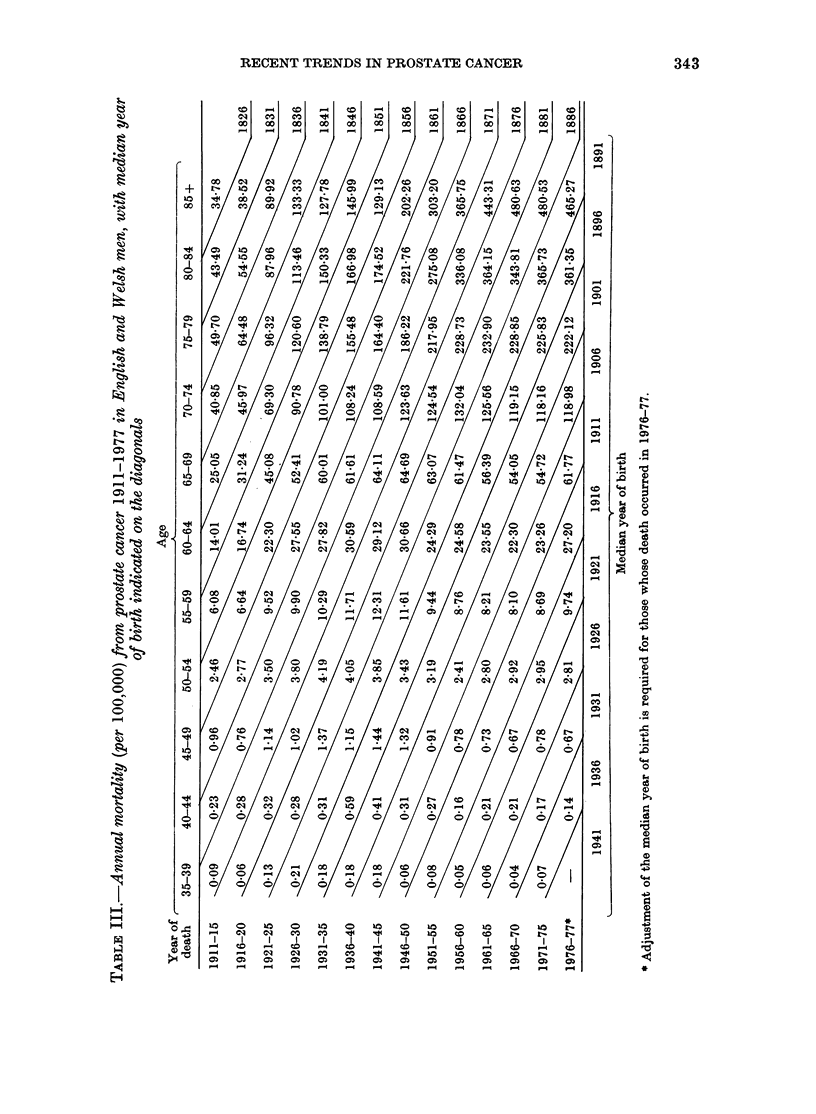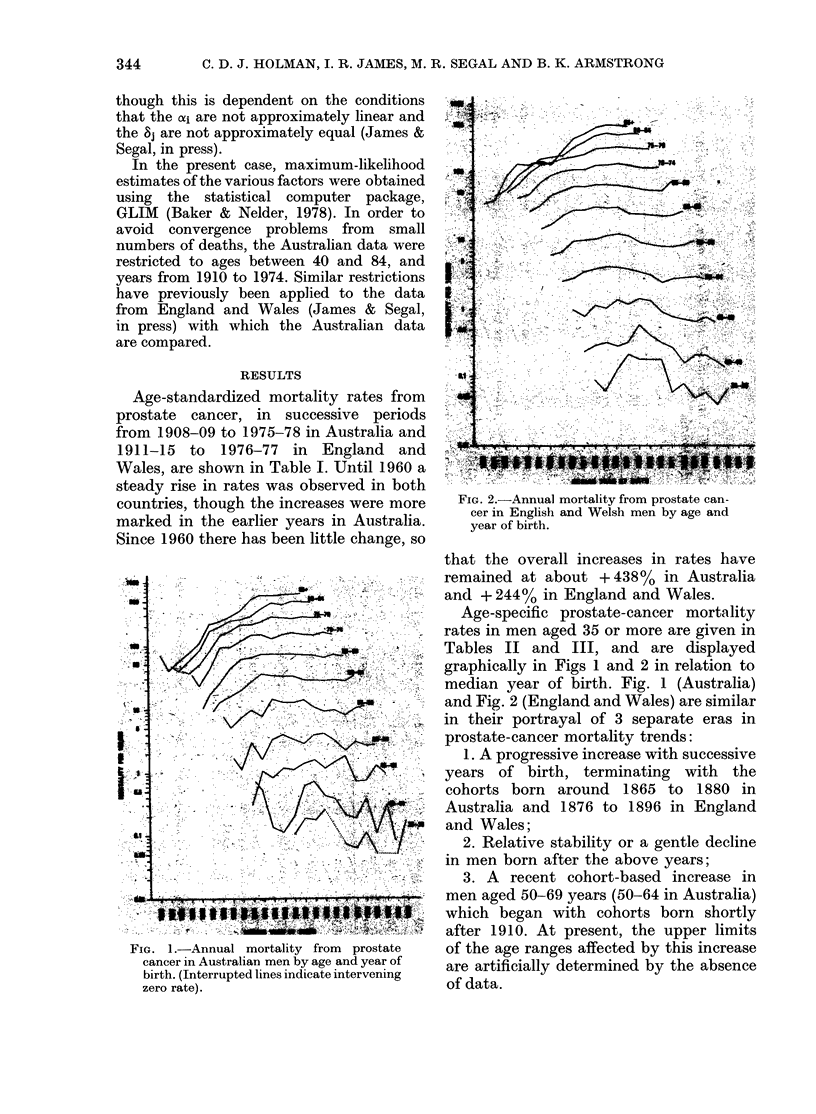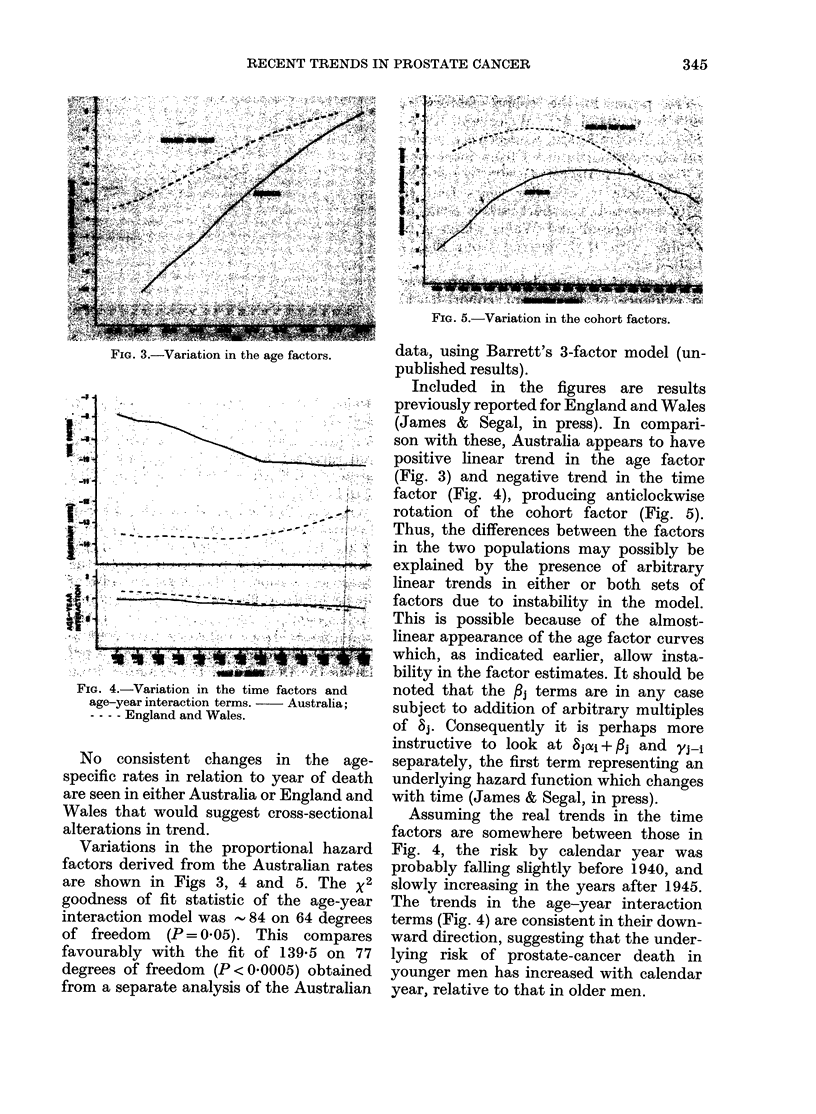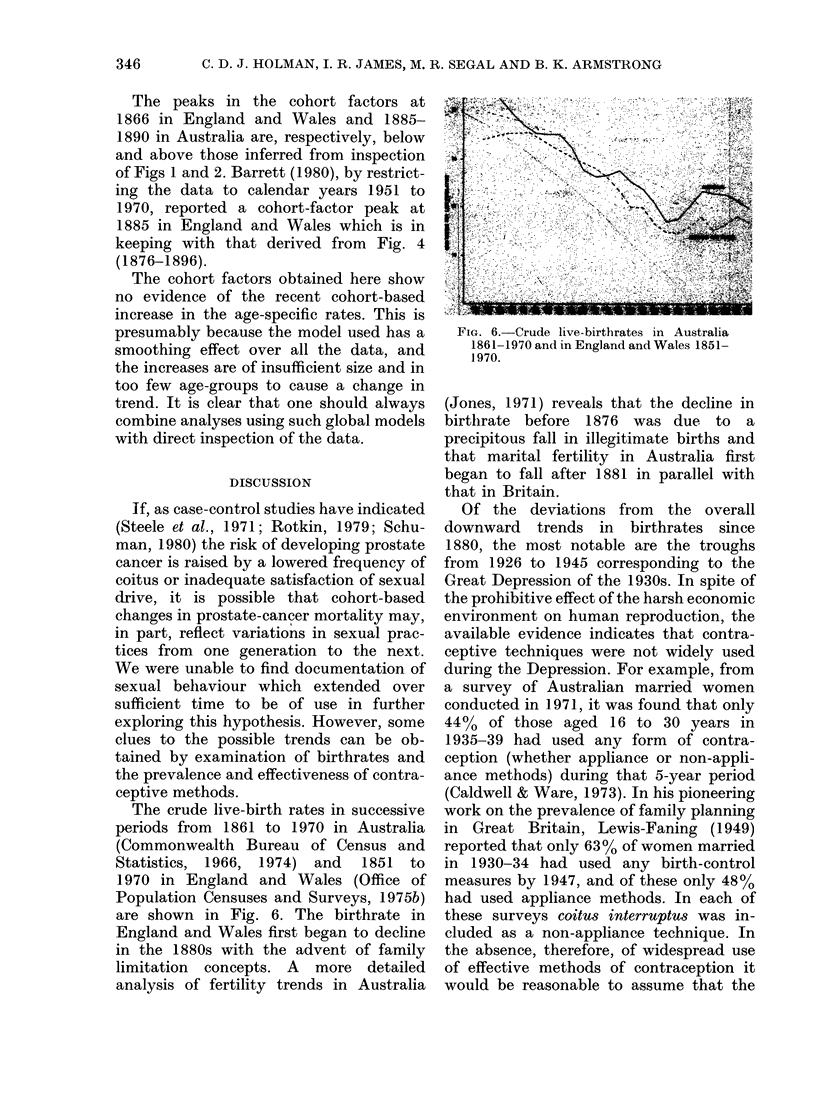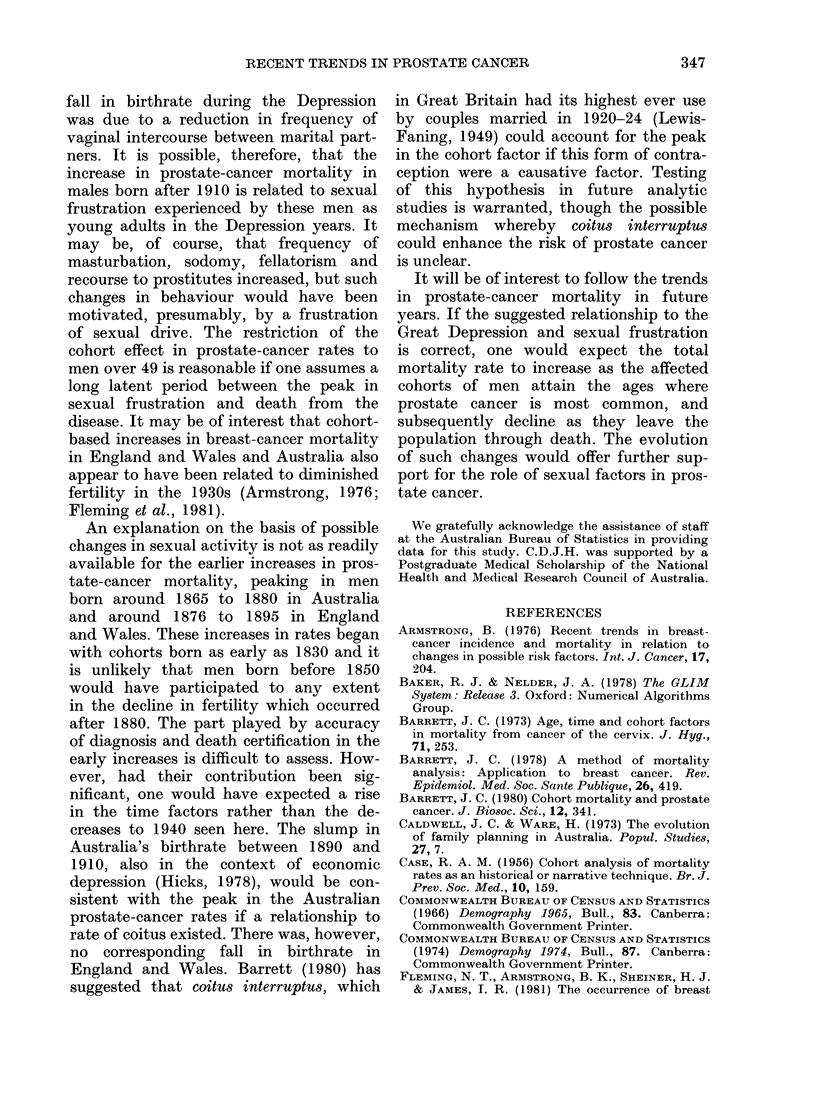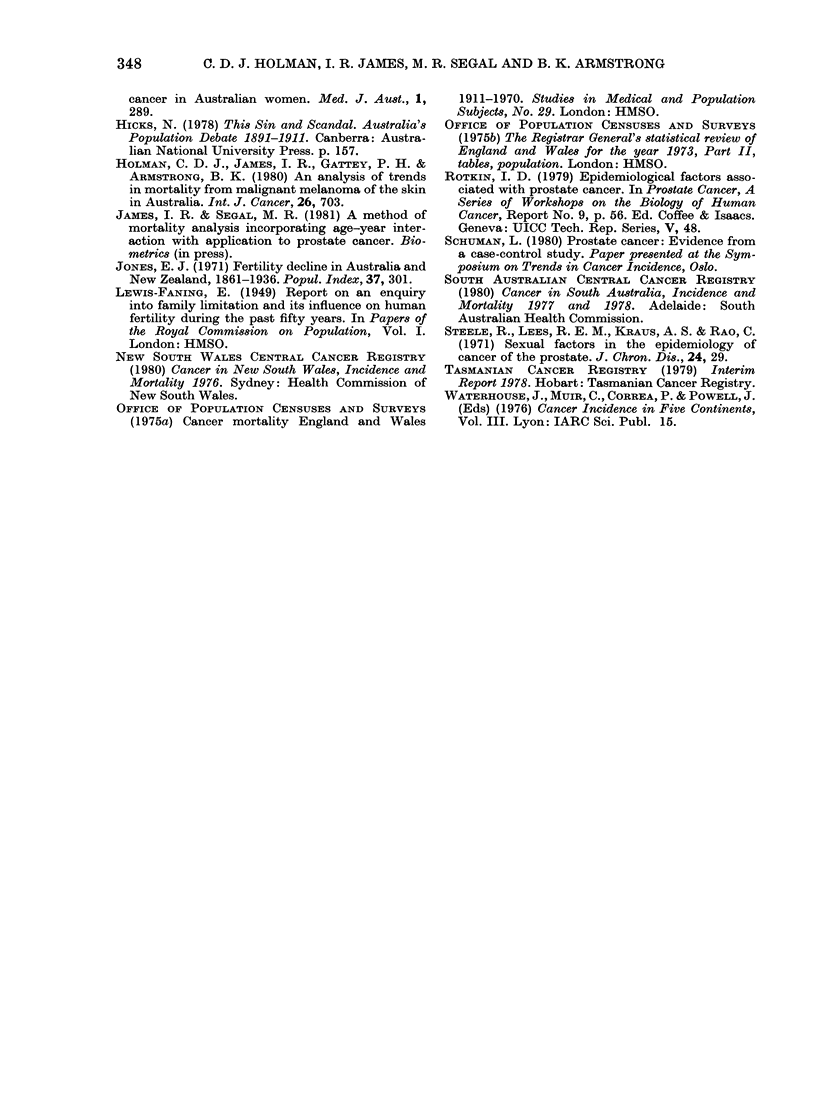# Recent trends in mortality from prostate cancer in male populations of Australia and England and Wales.

**DOI:** 10.1038/bjc.1981.190

**Published:** 1981-09

**Authors:** C. D. Holman, I. R. James, M. R. Segal, B. K. Armstrong

## Abstract

Mortality rates from cancer of the prostate in successive periods from 1908 to 1978 in Australia, and 1911 to 1977 in England and Wales, have been examined for trends with time and birth cohort. Age-specific rates and a proportional hazards model, designed to isolate the effect of birth cohort from those of calendar year and age, were used in the analysis. During the period of study, age-standardized mortality rose more than 5-fold in Australian men compared to just over 3-fold in men in England and Wales. In both countries the increases occurred almost entirely before 1960, with relative stability in age-standardized rates since then. The trends in mortality with year of birth were similar in the two sets of data. The risk of death from prostate cancer increased with successive birth cohorts to reach a peak in men born around 1865-1880 in Australia and men born around 1876-1896 in England and Wales. Males born later experienced a continuing reduction in rates, with the exception of age groups between 50 and 69 in which a further increase has appeared, starting with cohorts born after 1910. On the basis of current knowledge of the aetiology of prostate cancer, possible relationships between changes in sexual practices and prostate-cancer risk in successive generations have been explored. It is suggested that lowered sexual activity during the Great Depression may account for the recent cohort-based increases in mortality in middle-aged men.


*Br. J. Cancer* (1981) **44**, 340

### RECENT TRENDS IN MORTALITY FROM PROSTATE CANCER IN MALE POPULATIONS OF AUSTRALIA AND ENGLAND AND WALES

C. D. J. HOLMAN*, I. R. JAMES†, M. R. SEGAL† AND B. K. ARMSTRONG*

*From the *NH and MRC Research Unit in Epidemiology and Preventive Medicine and †Department of Mathematics, University of Western Australia*

Received 24 March 1981 Accepted 27 May 1981

**Summary.—Mortality rates from cancer of the prostate in successive periods from 1908 to 1978 in Australia, and 1911 to 1977 in England and Wales, have been examined for trends with time and birth cohort.**

**Age-specific rates and a proportional hazards model, designed to isolate the effect of birth cohort from those of calendar year and age, were used in the analysis.**

**During the period of study, age-standardized mortality rose more than 5-fold in Australian men compared to just over 3-fold in men in England and Wales. In both countries the increases occurred almost entirely before 1960, with relative stability in age-standardized rates since then.**

**The trends in mortality with year of birth were similar in the two sets of data. The risk of death from prostate cancer increased with successive birth cohorts to reach a peak in men born around 1865–1880 in Australia and men born around 1876–1896 in England and Wales. Males born later experienced a continuing reduction in rates, with the exception of age groups between 50 and 69 in which a further increase has appeared, starting with cohorts born after 1910.**

**On the basis of current knowledge of the aetiology of prostate cancer, possible relationships between changes in sexual practices and prostate-cancer risk in successive generations have been explored. It is suggested that lowered sexual activity during the Great Depression may account for the recent cohort-based increases in mortality in middle-aged men.**

OF THE 77 MALE POPULATIONS for which rates appear in *Cancer Incidence in Five Continents*, Vol. III (Waterhouse *et al.*, 1976), the prostate gland is the leading visceral cancer site in 9; second to lung or stomach in 25; and in a further 19 it ranks third after lung and stomach. In Australia (New South Wales Central Cancer Registry, 1980; South Australian Central Cancer Registry, 1980; Tasmanian Cancer Registry, 1979) prostate cancer is the second most frequent non-cutaneous malignancy in men, following cancer of the lung. In view of the relative importance of prostate cancer as a cause of morbidity, it is a paradox that its epidemiology has suffered comparative neglect. Nevertheless, clues to its relationships with endocrinological and sexual phenomena are now emerging, and in particular, male sexual frustration has appeared as a common factor (Steele *et al.*, 1971; Rotkin, 1979; Schuman, 1980).

It is our purpose here to describe the secular trends that have occurred in prostate cancer mortality in Australia and England and Wales, with emphasis on recent changes and their interpretation on the basis of associations found in analytic studies. At the same time, further elaboration on Barrett's model for mortality-trend analysis (Barrett, 1973, 1978) will

Correspondence and reprint requests to: Dr C. D. J. Holman, NH & MRC Research Unit in Epidemiology and Preventive Medicine, Department of Medicine, University of W.A., Crawley, 6008, Western Australia.

be made, with particular reference to the age–year interaction term proposed by James & Segal (in press).

### MATERIAL AND METHODS

Numbers of deaths from prostate cancer and estimates of the corresponding populations of men were obtained from the Australian Bureau of Statistics and the Office of Population Censuses and Surveys (1975*a*) of England and Wales. From the Australian data, which comprised annual deaths in 5-year age groups in years 1908 to 1978, age-specific mortality rates were calculated for each 5-year period from 1910–14 to 1970–74 and for the truncated periods, 1908–09 and 1975–78. The published data from England and Wales, also in 5-year age groups, were already grouped into 5-year periods, starting with 1911–15 and ending with 1966–70. In this form they have been the subject of two previous analyses (Barrett, 1980; James & Segal, in press), and on this occasion they were supplemented by data from 1971–75 and 1976–77. Apart from these differences in year groupings, the Australian and English-and-Welsh data were processed in the same manner.

In addition to direct examination of the age-specific rates in relation to year of birth (a variation on methods described by Case, 1956) age, time and cohort factors were estimated from the Australian data, by use of a proportional-hazards model which allows for the possibility of age–year interaction (James & Segal, in press). For a quinary–quinquennial table of mortality rates, this model takes the form,

$$\log E\,[p_{ij}] = \alpha_i\,\delta_j = \beta_j + \gamma_{j-i}$$

where E $[p_{ij}]$ is the expected rate in the (i,j)th cell; $\alpha_i$ is an age factor for the ith age group; $\beta_j$ and $\delta_j$ are, respectively, a time factor and an interaction term for the jth quinquennium and $\gamma_{j-i}$ is the cohort factor of the (j-i)th cohort. This model, which is an extension of that due to Barrett (1973, 1978) results when one assumes a hazard function of the form,

$$\lambda_{ij}\,(t) = \exp\,(\delta_j.a(t) + \beta_j + \gamma_{j-i})$$

where $\lambda_{ij}(t)$ is the hazard (*i.e.* risk of death) at any age t, given i and j and a(t) is some basic but undefined function of age which determines the relative risk of death. It should be noted that $\alpha_i$ is effectively a point estimate of a(t) based on a class interval of ages. The advantage of this model over earlier work (Barrett, 1973, 1978, 1980; Holman *et al.*, 1980) is that it not only allows the proportional effects of epoch of birth and epoch of death to enter multiplicatively into the hazard function, but it also allows the shape of the hazard to change with time, albeit in the fairly restrictive sense that the relative risk between two ages may change. This is reasonable, since a cross-sectional factor, although affecting all strata of the population at once, may still have differential effects according to age.

A major difficulty in the interpretation of age, time and cohort factor variation resulting from models of Barrett's 3-factor type is the possible inclusion in them of arbitrary linear trends (Barrett, 1978; Holman *et al.*, 1980). For example, for any c,

$$\begin{aligned}\log E\,[p_{ij}] &= \alpha_i + \beta_j + \gamma_{j-i}\\ &= (\alpha_i + ci) + (\beta_j - cj) + (\gamma_{j-i} + c(j\cdot i)).\end{aligned}$$

Thus, the addition of arbitrary trend to the age factor accompanied by subtraction of the trend from the time factor is compensated by a linear trend in the cohort factor. In this regard the age–year interaction model has the advantage of slightly greater stability,

TABLE I.—*Annual mortality (per* 100,000) *from prostate cancer in Australian men* 1908–1978 *and in English and Welsh men* 1911–1977

| Australia | | England and Wales | |
|---|---|---|---|
| Year of death | Age-standardized rates* | Year of death | Age-standardized rates* |
| 1908–09 | 2·94 (0·34) | | |
| 1910–14 | 2·68 (0·19) | 1911–15 | 3·72 (0·08) |
| 1915–19 | 4·05 (0·23) | 1916–20 | 4·43 (0·08) |
| 1920–24 | 6·49 (0·27) | 1921–25 | 6·55 (0·10) |
| 1925–29 | 8·69 (0·28) | 1926–30 | 8·08 (0·10) |
| 1930–34 | 10·91 (0·28) | 1931–35 | 9·05 (0·10) |
| 1935–39 | 12·52 (0·29) | 1936–40 | 9·81 (0·10) |
| 1940–44 | 12·67 (0·27) | 1941–45 | 9·95 (0·09) |
| 1945–49 | 13·16 (0·26) | 1946–50 | 11·10 (0·09) |
| 1950–54 | 14·88 (0·26) | 1951–55 | 11·75 (0·09) |
| 1955–59 | 15·79 (0·26) | 1956–60 | 12·51 (0·09) |
| 1960–64 | 15·07 (0·24) | 1961–65 | 12·66 (0·09) |
| 1965–69 | 15·45 (0·23) | 1966–70 | 12·35 (0·09) |
| 1970–74 | 15·80 (0·22) | 1971–75 | 12·52 (0·09) |
| 1975–78 | 15·81 (0·24) | 1976–77 | 12·81 (0·13) |

* Directly standardized to the age distribution of the Australian male population at the 1976 census. S.e. in parenthesis.

TABLE II.—*Annual mortality (per 100,000) from prostate cancer 1908–1978 in Australian men, with median year of birth indicated on the diagonals*

| Year of death | Age 35–39 | 40–44 | 45–49 | 50–54 | 55–59 | 60–64 | 65–69 | 70–74 | 75–79 | 80–84 | 85+ | Median year of birth (diagonal) |
|---|---|---|---|---|---|---|---|---|---|---|---|---|
| 1908–09* | — | 0·72 | — | 1·69 | 7·74 | 9·11 | 9·91 | 34·00 | 40·52 | 47·90 | 68·11 | |
| 1910–14 | — | 0·53 | 0·29 | 1·07 | 4·99 | 12·80 | 16·89 | 22·29 | 35·73 | 39·95 | 39·04 | 1820 |
| 1915–19 | — | 0·13 | 1·01 | 2·98 | 7·24 | 15·96 | 25·67 | 41·86 | 49·37 | 57·52 | 54·75 | 1825 |
| 1920–24 | 0·10 | 0·11 | 0·81 | 1·57 | 9·71 | 19·56 | 43·05 | 92·82 | 82·48 | 83·33 | 75·17 | 1830 |
| 1925–29 | 0·18 | 0·19 | 1·13 | 3·38 | 7·52 | 26·70 | 59·01 | 93·55 | 140·65 | 167·42 | 108·42 | 1835 |
| 1930–34 | 0·17 | 0·61 | 1·09 | 3·95 | 14·18 | 34·69 | 65·20 | 115·77 | 167·41 | 197·94 | 215·10 | 1840 |
| 1935–39 | 0·25 | 0·71 | 1·35 | 3·34 | 12·85 | 33·53 | 73·44 | 141·49 | 212·04 | 244·37 | 235·29 | 1845 |
| 1940–44 | — | 0·42 | 1·55 | 2·82 | 12·51 | 32·68 | 72·77 | 143·89 | 210·88 | 255·30 | 285·35 | 1850 |
| 1945–49 | 0·07 | 0·38 | 1·11 | 3·90 | 13·92 | 32·68 | 73·16 | 129·06 | 234·57 | 285·06 | 340·99 | 1855 |
| 1950–54 | 0·06 | 0·20 | 1·42 | 4·01 | 10·41 | 28·82 | 71·01 | 145·20 | 282·67 | 371·71 | 521·02 | 1860 |
| 1955–59 | 0·11 | 0·18 | 1·81 | 3·30 | 9·27 | 28·08 | 71·94 | 155·02 | 274·17 | 465·31 | 601·38 | 1865 |
| 1960–64 | 0·05 | 0·39 | 0·72 | 2·20 | 9·68 | 27·57 | 69·41 | 147·01 | 262·41 | 432·43 | 602·22 | 1870 |
| 1965–69 | — | 0·10 | 0·73 | 2·99 | 7·94 | 28·75 | 64·14 | 142·84 | 281·61 | 491·09 | 613·87 | 1875 |
| 1970–74 | 0·05 | 0·03 | 1·29 | 2·64 | 10·05 | 27·15 | 72·09 | 150·86 | 271·24 | 481·95 | 634·61 | 1880 |
| 1975–78* | 0·17 | 0·06 | 0·93 | 2·75 | 11·00 | 32·23 | 66·50 | 145·14 | 275·36 | 471·33 | 660·47 | 1885 |
| Median year of birth (diagonal) | | 1940 | 1935 | 1930 | 1925 | 1920 | 1915 | 1910 | 1905 | 1900 | 1895 | 1890 |

* Adjustment of the median year of birth is required for those whose death occurred in 1908–09 or 1975–78.

TABLE III.—*Annual mortality (per* 100,000) *from prostate cancer* 1911–1977 *in English and Welsh men, with median year of birth indicated on the diagonals*

| Year of death | Age 35–39 | 40–44 | 45–49 | 50–54 | 55–59 | 60–64 | 65–69 | 70–74 | 75–79 | 80–84 | 85+ | |
|---|---|---|---|---|---|---|---|---|---|---|---|---|
| 1911–15 | 0·09 | 0·23 | 0·96 | 2·46 | 6·08 | 14·01 | 25·05 | 40·85 | 49·70 | 43·49 | 34·78 | |
| 1916–20 | 0·06 | 0·28 | 0·76 | 2·77 | 6·64 | 16·74 | 31·24 | 45·97 | 64·48 | 54·55 | 38·52 | 1826 |
| 1921–25 | 0·13 | 0·32 | 1·14 | 3·50 | 9·52 | 22·30 | 45·08 | 69·30 | 96·32 | 87·96 | 89·92 | 1831 |
| 1926–30 | 0·21 | 0·28 | 1·02 | 3·80 | 9·90 | 27·55 | 52·41 | 90·78 | 120·60 | 113·46 | 133·33 | 1836 |
| 1931–35 | 0·18 | 0·31 | 1·37 | 4·19 | 10·29 | 27·82 | 60·01 | 101·00 | 138·79 | 150·33 | 127·78 | 1841 |
| 1936–40 | 0·18 | 0·59 | 1·15 | 4·05 | 11·71 | 30·59 | 61·61 | 108·24 | 155·48 | 166·98 | 145·99 | 1846 |
| 1941–45 | 0·18 | 0·41 | 1·44 | 3·85 | 12·31 | 29·12 | 64·11 | 108·59 | 164·40 | 174·52 | 129·13 | 1851 |
| 1946–50 | 0·06 | 0·31 | 1·32 | 3·43 | 11·61 | 30·66 | 64·69 | 123·63 | 186·22 | 221·76 | 202·26 | 1856 |
| 1951–55 | 0·08 | 0·27 | 0·91 | 3·19 | 9·44 | 24·29 | 63·07 | 124·54 | 217·95 | 275·08 | 303·20 | 1861 |
| 1956–60 | 0·05 | 0·16 | 0·78 | 2·41 | 8·76 | 24·58 | 61·47 | 132·04 | 228·73 | 336·08 | 365·75 | 1866 |
| 1961–65 | 0·06 | 0·21 | 0·73 | 2·80 | 8·21 | 23·55 | 56·39 | 125·56 | 232·90 | 364·15 | 443·31 | 1871 |
| 1966–70 | 0·04 | 0·21 | 0·67 | 2·92 | 8·10 | 22·30 | 54·05 | 119·15 | 228·85 | 343·81 | 480·63 | 1876 |
| 1971–75 | 0·07 | 0·17 | 0·78 | 2·95 | 8·69 | 23·26 | 54·72 | 118·16 | 225·83 | 365·73 | 480·53 | 1881 |
| 1976–77* | — | 0·14 | 0·67 | 2·81 | 9·74 | 27·20 | 61·77 | 118·98 | 222·12 | 361·35 | 465·27 | 1886 |
| Median year of birth | 1941 | 1936 | 1931 | 1926 | 1921 | 1916 | 1911 | 1906 | 1901 | 1896 | 1891 | |

* Adjustment of the median year of birth is required for those whose death occurred in 1976–77.

though this is dependent on the conditions that the $\alpha_i$ are not approximately linear and the $\delta_j$ are not approximately equal (James & Segal, in press).

In the present case, maximum-likelihood estimates of the various factors were obtained using the statistical computer package, GLIM (Baker & Nelder, 1978). In order to avoid convergence problems from small numbers of deaths, the Australian data were restricted to ages between 40 and 84, and years from 1910 to 1974. Similar restrictions have previously been applied to the data from England and Wales (James & Segal, in press) with which the Australian data are compared.

### RESULTS

Age-standardized mortality rates from prostate cancer, in successive periods from 1908–09 to 1975–78 in Australia and 1911–15 to 1976–77 in England and Wales, are shown in Table I. Until 1960 a steady rise in rates was observed in both countries, though the increases were more marked in the earlier years in Australia. Since 1960 there has been little change, so that the overall increases in rates have remained at about +438% in Australia and +244% in England and Wales.

FIG. 1.—Annual mortality from prostate cancer in Australian men by age and year of birth. (Interrupted lines indicate intervening zero rate).

FIG. 2.—Annual mortality from prostate cancer in English and Welsh men by age and year of birth.

Age-specific prostate-cancer mortality rates in men aged 35 or more are given in Tables II and III, and are displayed graphically in Figs 1 and 2 in relation to median year of birth. Fig. 1 (Australia) and Fig. 2 (England and Wales) are similar in their portrayal of 3 separate eras in prostate-cancer mortality trends:

1. A progressive increase with successive years of birth, terminating with the cohorts born around 1865 to 1880 in Australia and 1876 to 1896 in England and Wales;

2. Relative stability or a gentle decline in men born after the above years;

3. A recent cohort-based increase in men aged 50–69 years (50–64 in Australia) which began with cohorts born shortly after 1910. At present, the upper limits of the age ranges affected by this increase are artificially determined by the absence of data.

FIG. 3.—Variation in the age factors.

FIG. 4.—Variation in the time factors and age–year interaction terms. —— Australia; - - - - England and Wales.

No consistent changes in the age-specific rates in relation to year of death are seen in either Australia or England and Wales that would suggest cross-sectional alterations in trend.

Variations in the proportional hazard factors derived from the Australian rates are shown in Figs 3, 4 and 5. The $\chi^2$ goodness of fit statistic of the age-year interaction model was $\sim 84$ on 64 degrees of freedom ($P = 0{\cdot}05$). This compares favourably with the fit of 139·5 on 77 degrees of freedom ($P < 0{\cdot}0005$) obtained from a separate analysis of the Australian

FIG. 5.—Variation in the cohort factors.

data, using Barrett's 3-factor model (unpublished results).

Included in the figures are results previously reported for England and Wales (James & Segal, in press). In comparison with these, Australia appears to have positive linear trend in the age factor (Fig. 3) and negative trend in the time factor (Fig. 4), producing anticlockwise rotation of the cohort factor (Fig. 5). Thus, the differences between the factors in the two populations may possibly be explained by the presence of arbitrary linear trends in either or both sets of factors due to instability in the model. This is possible because of the almost-linear appearance of the age factor curves which, as indicated earlier, allow instability in the factor estimates. It should be noted that the $\beta_j$ terms are in any case subject to addition of arbitrary multiples of $\delta_j$. Consequently it is perhaps more instructive to look at $\delta_j\alpha_i + \beta_j$ and $\gamma_{j-i}$ separately, the first term representing an underlying hazard function which changes with time (James & Segal, in press).

Assuming the real trends in the time factors are somewhere between those in Fig. 4, the risk by calendar year was probably falling slightly before 1940, and slowly increasing in the years after 1945. The trends in the age–year interaction terms (Fig. 4) are consistent in their downward direction, suggesting that the underlying risk of prostate-cancer death in younger men has increased with calendar year, relative to that in older men.

The peaks in the cohort factors at 1866 in England and Wales and 1885–1890 in Australia are, respectively, below and above those inferred from inspection of Figs 1 and 2. Barrett (1980), by restricting the data to calendar years 1951 to 1970, reported a cohort-factor peak at 1885 in England and Wales which is in keeping with that derived from Fig. 4 (1876–1896).

The cohort factors obtained here show no evidence of the recent cohort-based increase in the age-specific rates. This is presumably because the model used has a smoothing effect over all the data, and the increases are of insufficient size and in too few age-groups to cause a change in trend. It is clear that one should always combine analyses using such global models with direct inspection of the data.

### DISCUSSION

If, as case-control studies have indicated (Steele *et al.*, 1971; Rotkin, 1979; Schuman, 1980) the risk of developing prostate cancer is raised by a lowered frequency of coitus or inadequate satisfaction of sexual drive, it is possible that cohort-based changes in prostate-cancer mortality may, in part, reflect variations in sexual practices from one generation to the next. We were unable to find documentation of sexual behaviour which extended over sufficient time to be of use in further exploring this hypothesis. However, some clues to the possible trends can be obtained by examination of birthrates and the prevalence and effectiveness of contraceptive methods.

The crude live-birth rates in successive periods from 1861 to 1970 in Australia (Commonwealth Bureau of Census and Statistics, 1966, 1974) and 1851 to 1970 in England and Wales (Office of Population Censuses and Surveys, 1975*b*) are shown in Fig. 6. The birthrate in England and Wales first began to decline in the 1880s with the advent of family limitation concepts. A more detailed analysis of fertility trends in Australia

FIG. 6.—Crude live-birthrates in Australia 1861–1970 and in England and Wales 1851–1970.

(Jones, 1971) reveals that the decline in birthrate before 1876 was due to a precipitous fall in illegitimate births and that marital fertility in Australia first began to fall after 1881 in parallel with that in Britain.

Of the deviations from the overall downward trends in birthrates since 1880, the most notable are the troughs from 1926 to 1945 corresponding to the Great Depression of the 1930s. In spite of the prohibitive effect of the harsh economic environment on human reproduction, the available evidence indicates that contraceptive techniques were not widely used during the Depression. For example, from a survey of Australian married women conducted in 1971, it was found that only 44% of those aged 16 to 30 years in 1935–39 had used any form of contraception (whether appliance or non-appliance methods) during that 5-year period (Caldwell & Ware, 1973). In his pioneering work on the prevalence of family planning in Great Britain, Lewis-Faning (1949) reported that only 63% of women married in 1930–34 had used any birth-control measures by 1947, and of these only 48% had used appliance methods. In each of these surveys *coitus interruptus* was included as a non-appliance technique. In the absence, therefore, of widespread use of effective methods of contraception it would be reasonable to assume that the fall in birthrate during the Depression was due to a reduction in frequency of vaginal intercourse between marital partners. It is possible, therefore, that the increase in prostate-cancer mortality in males born after 1910 is related to sexual frustration experienced by these men as young adults in the Depression years. It may be, of course, that frequency of masturbation, sodomy, fellatorism and recourse to prostitutes increased, but such changes in behaviour would have been motivated, presumably, by a frustration of sexual drive. The restriction of the cohort effect in prostate-cancer rates to men over 49 is reasonable if one assumes a long latent period between the peak in sexual frustration and death from the disease. It may be of interest that cohort-based increases in breast-cancer mortality in England and Wales and Australia also appear to have been related to diminished fertility in the 1930s (Armstrong, 1976; Fleming *et al.*, 1981).

An explanation on the basis of possible changes in sexual activity is not as readily available for the earlier increases in prostate-cancer mortality, peaking in men born around 1865 to 1880 in Australia and around 1876 to 1895 in England and Wales. These increases in rates began with cohorts born as early as 1830 and it is unlikely that men born before 1850 would have participated to any extent in the decline in fertility which occurred after 1880. The part played by accuracy of diagnosis and death certification in the early increases is difficult to assess. However, had their contribution been significant, one would have expected a rise in the time factors rather than the decreases to 1940 seen here. The slump in Australia's birthrate between 1890 and 1910, also in the context of economic depression (Hicks, 1978), would be consistent with the peak in the Australian prostate-cancer rates if a relationship to rate of coitus existed. There was, however, no corresponding fall in birthrate in England and Wales. Barrett (1980) has suggested that *coitus interruptus*, which in Great Britain had its highest ever use by couples married in 1920–24 (Lewis-Faning, 1949) could account for the peak in the cohort factor if this form of contraception were a causative factor. Testing of this hypothesis in future analytic studies is warranted, though the possible mechanism whereby *coitus interruptus* could enhance the risk of prostate cancer is unclear.

It will be of interest to follow the trends in prostate-cancer mortality in future years. If the suggested relationship to the Great Depression and sexual frustration is correct, one would expect the total mortality rate to increase as the affected cohorts of men attain the ages where prostate cancer is most common, and subsequently decline as they leave the population through death. The evolution of such changes would offer further support for the role of sexual factors in prostate cancer.

We gratefully acknowledge the assistance of staff at the Australian Bureau of Statistics in providing data for this study. C.D.J.H. was supported by a Postgraduate Medical Scholarship of the National Health and Medical Research Council of Australia.

### REFERENCES

Armstrong, B. (1976) Recent trends in breast-cancer incidence and mortality in relation to changes in possible risk factors. *Int. J. Cancer*, **17**, 204.

Baker, R. J. & Nelder, J. A. (1978) *The GLIM System: Release 3*. Oxford: Numerical Algorithms Group.

Barrett, J. C. (1973) Age, time and cohort factors in mortality from cancer of the cervix. *J. Hyg.*, **71**, 253.

Barrett, J. C. (1978) A method of mortality analysis: Application to breast cancer. *Rev. Epidemiol. Med. Soc. Sante Publique*, **26**, 419.

Barrett, J. C. (1980) Cohort mortality and prostate cancer. *J. Biosoc. Sci.*, **12**, 341.

Caldwell, J. C. & Ware, H. (1973) The evolution of family planning in Australia. *Popul. Studies*, **27**, 7.

Case, R. A. M. (1956) Cohort analysis of mortality rates as an historical or narrative technique. *Br. J. Prev. Soc. Med.*, **10**, 159.

Commonwealth Bureau of Census and Statistics (1966) *Demography 1965*, Bull., **83**. Canberra: Commonwealth Government Printer.

Commonwealth Bureau of Census and Statistics (1974) *Demography 1974*, Bull., **87**. Canberra: Commonwealth Government Printer.

Fleming, N. T., Armstrong, B. K., Sheiner, H. J. & James, I. R. (1981) The occurrence of breast cancer in Australian women. *Med. J. Aust.*, **1**, 289.

HICKS, N. (1978) *This Sin and Scandal. Australia's Population Debate 1891–1911.* Canberra: Australian National University Press. p. 157.

HOLMAN, C. D. J., JAMES, I. R., GATTEY, P. H. & ARMSTRONG, B. K. (1980) An analysis of trends in mortality from malignant melanoma of the skin in Australia. *Int. J. Cancer*, **26**, 703.

JAMES, I. R. & SEGAL, M. R. (1981) A method of mortality analysis incorporating age–year interaction with application to prostate cancer. *Biometrics* (in press).

JONES, E. J. (1971) Fertility decline in Australia and New Zealand, 1861–1936. *Popul. Index*, **37**, 301.

LEWIS-FANING, E. (1949) Report on an enquiry into family limitation and its influence on human fertility during the past fifty years. In *Papers of the Royal Commission on Population*, Vol. I. London: HMSO.

NEW SOUTH WALES CENTRAL CANCER REGISTRY (1980) *Cancer in New South Wales, Incidence and Mortality 1976.* Sydney: Health Commission of New South Wales.

OFFICE OF POPULATION CENSUSES AND SURVEYS (1975*a*) Cancer mortality England and Wales 1911–1970. *Studies in Medical and Population Subjects, No. 29.* London: HMSO.

OFFICE OF POPULATION CENSUSES AND SURVEYS (1975*b*) *The Registrar General's statistical review of England and Wales for the year 1973, Part II, tables, population.* London: HMSO.

ROTKIN, I. D. (1979) Epidemiological factors associated with prostate cancer. In *Prostate Cancer, A Series of Workshops on the Biology of Human Cancer*, Report No. 9, p. 56. Ed. Coffee & Isaacs. Geneva: UICC Tech. Rep. Series, **V**, 48.

SCHUMAN, L. (1980) Prostate cancer: Evidence from a case-control study. *Paper presented at the Symposium on Trends in Cancer Incidence, Oslo.*

SOUTH AUSTRALIAN CENTRAL CANCER REGISTRY (1980) *Cancer in South Australia, Incidence and Mortality 1977 and 1978.* Adelaide: South Australian Health Commission.

STEELE, R., LEES, R. E. M., KRAUS, A. S. & RAO, C. (1971) Sexual factors in the epidemiology of cancer of the prostate. *J. Chron. Dis.*, **24**, 29.

TASMANIAN CANCER REGISTRY (1979) *Interim Report 1978.* Hobart: Tasmanian Cancer Registry.

WATERHOUSE, J., MUIR, C., CORREA, P. & POWELL, J. (Eds) (1976) *Cancer Incidence in Five Continents*, Vol. III. Lyon: IARC Sci. Publ. 15.